# A microRNA Arising from the Negative Strand of SARS-CoV-2 Genome Targets FOS to Reduce AP-1 Activity

**DOI:** 10.3390/ncrna9030033

**Published:** 2023-05-23

**Authors:** Francesco Greco, Elisa Lorefice, Claudia Carissimi, Ilaria Laudadio, Fabiola Ciccosanti, Martina Di Rienzo, Francesca Colavita, Silvia Meschi, Fabrizio Maggi, Gian Maria Fimia, Valerio Fulci

**Affiliations:** 1 Dipartimento di Medicina Molecolare, Università di Roma “La Sapienza”, 00161 Rome, Italy; 2 Department of Epidemiology, Preclinical Research and Advanced Diagnostics, National Institute for Infectious Diseases IRCCS ‘L. Spallanzani’, 00149 Rome, Italy; 3 Istituto Pasteur Italia—Fondazione Cenci Bolognetti, 00161 Rome, Italy

**Keywords:** microRNA, SARS-CoV-2, FOS, AP-1, SARS-CoV-2-miR-AS1

## Abstract

Virus-encoded microRNAs were first reported in the Epstein–Barr virus in 2004. Subsequently, a few hundred viral miRNAs have been identified, mainly in DNA viruses belonging to the *herpesviridae* family. To date, only 30 viral miRNAs encoded by RNA viruses are reported by miRBase. Since the outbreak of the SARS-CoV-2 pandemic, several studies have predicted and, in some cases, experimentally validated miRNAs originating from the positive strand of the SARS-CoV-2 genome. By integrating NGS data analysis and qRT-PCR approaches, we found that SARS-CoV-2 also encodes for a viral miRNA arising from the minus (antisense) strand of the viral genome, in the region encoding for ORF1ab, herein referred to as SARS-CoV-2-miR-AS1. Our data show that the expression of this microRNA increases in a time course analysis of SARS-CoV-2 infected cells. Furthermore, enoxacin treatment enhances the accumulation of the mature SARS-CoV-2-miR-AS1 in SARS-CoV-2 infected cells, arguing for a Dicer-dependent processing of this small RNA. In silico analysis suggests that SARS-CoV-2-miR-AS1 targets a set of genes which are translationally repressed during SARS-CoV-2 infection. We experimentally validated that SARS-CoV-2-miR-AS1 targets FOS, thus repressing the AP-1 transcription factor activity in human cells.

## 1. Introduction

Virus-encoded miRNAs were first reported in Epstein–Barr virus [1]. Subsequently, a few hundred virus-encoded miRNAs have been identified, mainly in DNA viruses belonging to the *herpesviridae* family [2]. Although some computational tools to identify virus-encoded miRNAs have been developed and exploited [3,4], currently the major route to the discovery of novel viral miRNAs is the analysis of small RNA-seq data obtained from virus-infected cells or tissues [5].

The vast majority of known viral miRNAs are encoded by DNA viruses [6]. It has been highlighted that RNA genomes containing miRNA hairpins are likely to be cleaved by Drosha, thus preventing genome packaging into new viral particles. These considerations possibly explain the small number of miRNAs encoded by RNA viruses identified thus far [5]. Furthermore, the biogenesis of miRNAs encoded by cytoplasmic RNA viruses has long been considered puzzling, as Drosha, the enzyme required for pre-miRNA hairpin cleavage, is localized in the nucleus of eukaryotic cells [7]. However, later findings have highlighted that in vertebrates as well as in arthropods, Drosha is relocalized to the cytoplasm upon RNA virus infection to act in an interferon-independent, RNAi-mediated antiviral response [8]. These data provide a rationale explaining the previously reported processing of miRNA hairpins artificially introduced in cytoplasmic RNA viral vectors [9]. To date, 30 miRNAs encoded by RNA viruses are reported by miRBase [6,10].

SARS-CoV-2 belongs to the beta-coronavirus family and has a complex life cycle which implies the synthesis of both positive and negative polarity RNA molecules [11]. Positive polarity RNA molecules include the full-length genome, which is also used as a mRNA to drive ORF1ab translation, and a set of sub-genomic RNAs (sgRNAs) serving as mRNAs to drive the synthesis of the viral proteins. The production of sgRNAs proceeds first through the discontinuous transcription of the positive genome to yield negative polarity sgRNAs. Subsequently each of these negative strand sgRNAs acts as a template for the synthesis of the corresponding positive polarity sgRNA. Finally, a full-length negative polarity genomic strand is transcribed and used as a template to replicate the original positive strand genome, which will be packaged into new viral particles.

Recently, it has been shown that SARS-CoV-1 and SARS-CoV-2 encode for small viral RNAs [12,13]. Recent reports have highlighted that SARS-CoV-2 plus RNA strand is processed to yield a microRNA (miR-O7a) which negatively regulates the interferon pathway [14,15]. Here, we report that SARS-CoV-2 encodes for a further miRNA arising from the full-length minus strand viral genomic RNA; this viral miRNA targets the human mRNA encoding for c-FOS protein, thus repressing AP-1 activity in human cells.

## 2. Results

### 2.1. Identification of a Novel microRNA Encoded by SARS-CoV-2 Negative Strand Genomic RNA

To identify putative miRNAs encoded by SARS-CoV-2, we took advantage of publicly available miRNA-seq datasets obtained from Calu3 cells infected with SARS-CoV-2 [16]. Briefly, reads were trimmed and aligned to the human genome to discard all reads corresponding to small RNAs encoded by the human genome. All reads that failed to align to the human genome were then aligned to the SARS-CoV-2 genome. As several negative polarity RNA molecules are synthesized during the life cycle of SARS-CoV-2, it is conceivable that a subset of these RNA molecules could be processed to yield mature miRNAs. We, therefore, allowed alignment to both the plus and minus strands of SARS-CoV-2. We then looked for clusters of overlapping reads mapping to the SARS-CoV-2 genome, resembling the typical coverage pattern of miRNAs. Our analysis highlighted 80 regions of the SARS-CoV-2 genome giving rise to discrete clusters of overlapping, 19~25 nt long reads, a pattern which is a hallmark of miRNAs.

**Figure 1:**
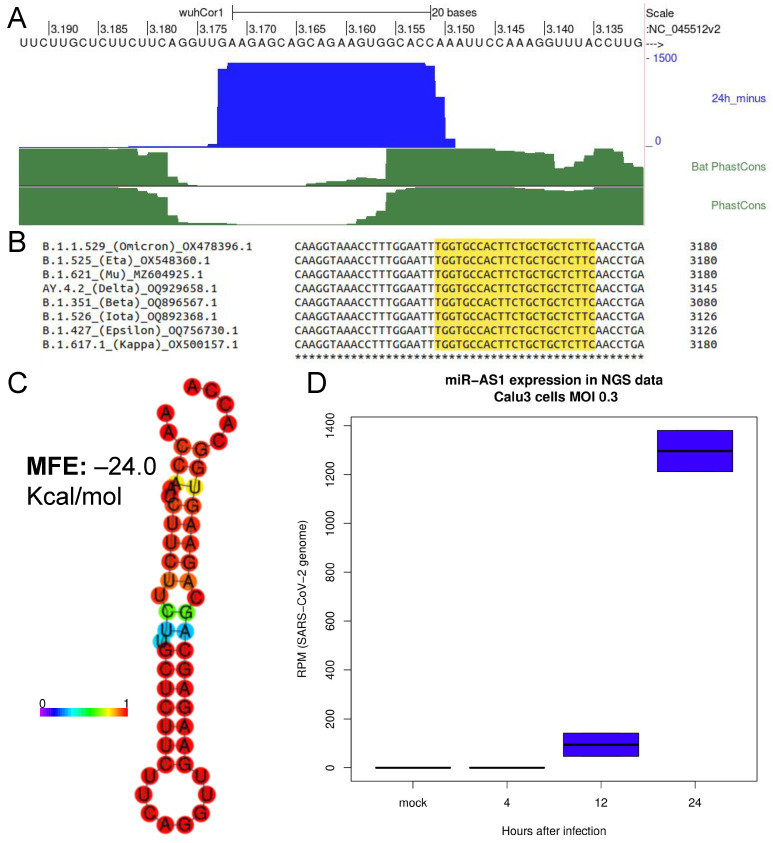
NGS data analysis unveils a novel putative miRNA encoded by SARS-CoV-2 minus strand. (**A**): miR-AS1 locus on the UCSC genome browser. Blue track: miRNA-seq coverage (negative strand) normalized by SARS-CoV-2 mapping reads (RPM) at 24 h after infection of Calu-3 cells (GEO GSE148729 data). Green tracks: phastCons score across 44 bat coronaviruses (Bat Phastcons) and 119 vertebrate coronaviruses (Phastcons). (**B**): Alignment of major SARS-CoV-2 lineages shows full conservation of miR-AS1 sequence (highlighted in yellow). Asterisks (*) denote fully coserved nucleotides. (**C**): putative structure of pre-miR-AS1 computed using RNAfold. Color scale: base-pair probabilities. (**D**): Time course of miR-AS1 expression in Calu-3 cells (GEO GSE148729 data).

We used RNAfold [17] to verify whether the neighboring RNA region of each candidate miRNA could fold into a pre-miRNA-like hairpin. We selected candidates lying in a single stem of the predicted structure with at least 60% of the mature miRNA residues paired. We arbitrarily set a MFE cutoff of −20 Kcal/mol to select putative miRNAs for further validation. These criteria resulted in the selection of four putative miRNA. We next focused our attention on a candidate viral microRNA which showed a tenfold higher read coverage compared to each of the other three candidates. This putative miRNA lies at nt 3151–3173 on the minus (Antisense) strand of the SARS-CoV-2 genome, complementary to the region of the viral genome encoding for ORF1ab (Figure 1A). We, therefore, will refer to this miRNA as SARS-CoV-2-miR-AS1 (miR-AS1). Interestingly, Phastcons scores for bat coronaviruses and vertebrate coronaviruses clearly show that this region corresponds to a very poorly conserved region of SARS-CoV-2 (Figure 1A, green tracks). On the other hand, the alignment of the SARS-CoV-2 genomes belonging to the major Pango lineages highlights that miR-AS1 and the surrounding region are conserved across all SARS-CoV-2 lineages. Our RNAfold analysis computed a secondary pre-miR-like structure for the putative pre-miR-AS1 RNA with an estimated MFE of −24 Kcal/mol (Figure 1B). Furthermore, miRNA-seq data in Calu3 cells highlight that miR-AS1 reads progressively accumulate during SARS-CoV-2 infection (Figure 1C). Time course data suggest that miR-AS1 expression occurs at the late stages of the infection.

**Figure 2:**
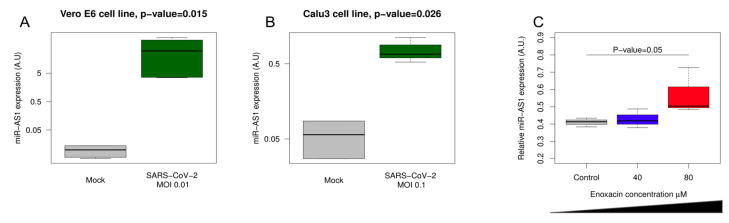
miR-AS1 is specifically expressed in SARS-CoV-2 infected cells and its expression is enhanced by enoxacin. qPCR validation of miR-AS1 expression in infected Vero E6 cells (panel (**A**)) and Calu-3 cells (panel (**B**)). (**C**): Enoxacin treatment enhances miR-AS1 processing in Calu-3 cells.

To further confirm with an alternative technique that miR-AS1 is specifically expressed in SARS-CoV-2 infected cells, we designed qPCR primers to amplify miR-AS1. As a negative control to ensure that the signal detected was not merely due to the amplification of longer RNAs corresponding to the negative strand of the SARS-CoV-2 genome, we designed primers to amplify a nearby (~200 nucleotides upstream miR-AS1) control region of the negative strand of the viral genome where no miRNA-like reads were identified (Supplementary Figure S1A), but which was predicted to fold into a secondary structure comparable to the one of the putative miR-AS1 precursor (Figure 1B, Supplementary Figure S1B). Our data reveal a significant induction of miR-AS1 in both Vero E6 cells (MOI 0.01) and Calu3 cells (MOI 0.1) infected with SARS-CoV-2 as compared to non-infected cells (Figure 2A,B). On the contrary, control primers did not yield any detectable amplification (Supplementary Figure S1C, blue line), indicating that not only miR-AS1 primers amplified the mature miRNA and not the negative strand of the viral genome, but also that miR-AS1 expression specifically arises from SARS-CoV-2 infection in different cell lines.

### 2.2. Promoting miRNA Processing Increases the Expression of SARS-CoV-2 miR-AS1

To verify whether miR-AS1 is processed by the microRNA processing pathway in human cells, we performed SARS-CoV-2 infection in enoxacin-treated cells. Enoxacin has been previously shown to promote the processing of miRNAs [18,19] as well as other Dicer-processed small RNAs [20].

Enoxacin was previously reported to mildly affect SARS-CoV-2 replication (EC_50_ = 120 μM) [21]. Indeed, although we used lower concentrations (40 μM to 80 μM), we observed a mild yet significant effect of enoxacin on the viral replication as assessed by qPCR using primers matching the RdRP gene encoded by SARS-CoV-2 (Supplementary Figure S2). Therefore, in order to account for the effect of enoxacin on SARS-CoV-2 replication, we normalized miR-AS1 expression in enoxacin-treated cells on the viral RNA load. Our data show that increasing concentrations of enoxacin result in increasing accumulation of mature SARS-CoV-2 miR-AS1, supporting that biogenesis of miR-AS1 is driven by the endogenous miRNA processing machinery (Figure 2C). Altogether, these data confirm that miR-AS1 is not a RNA degradation byproduct but rather a microRNA-like small RNA processed by the canonical miRNA processing pathway.

### 2.3. A Set of Genes Translationally Repressed during SARS-CoV-2 Infection Is Significantly Enriched for miR-AS1 Seed Matches

**Figure 3:**
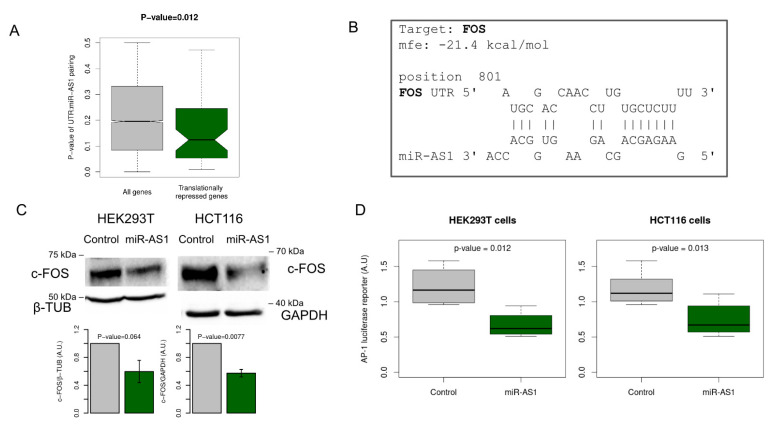
miR-AS1 targets FOS and repressed AP-1 activity. (**A**): Genes that are translationally repressed during SARS-CoV-2 infection display significantly better pairing to miR-AS1 “seed” compared to all other genes. (**B**): miR-AS1 pairing with FOS 3′ UTR as assessed using RNAhybrid. (**C**): Western blot analysis of c-FOS protein in HEK293T and HCT116 cells transfected with control siRNA or miR-AS1. (**D**): miR-AS1 represses AP-1 activity in HEK293T (left panel, n = 4) and HCT116 (right panel, n = 4) cells.

In order to highlight the role of miR-AS1 during SARS-CoV-2 infection, we looked for miR-AS1 putative targets in human cells. Since miRNAs act by post-transcriptionally modulating gene expression, we chose to focus on host genes which are translationally repressed during SARS-CoV-2 infection as assessed by the ribosome (Ribo)-seq [22]. We focused on the set of 121 genes reported to have a “Translational Enhancement” score <−1 in human bronchial epithelial cells at 96 h post-infection. Taking advantage of the RNAhybrid software [23], we looked for putative miR-AS1 target sites in the 3′ UTRs of the 121 translationally repressed genes (Supplementary Table S2). As a control, the same analysis was performed for all other genes. When we compared the distribution of the *p*-values for the targeting by miR-AS1 we found that genes undergoing a translational repression during SARS-CoV-2 infection display significantly lower *p*-values (Figure 3A). This observation suggests that host genes which are translationally repressed during SARS-CoV-2 infection may contain *bona fide* targets of miR-AS1.

### 2.4. miR-AS1 Is a Negative Modulator of AP-1 Transcription Factor via Targeting FOS

FOS and JUN, key components of the AP-1 transcription factor, are among the above-mentioned set of 121 translationally repressed genes during SARS-CoV-2 infection. AP-1 is a key mediator of the immune response and several reports have highlighted its induction upon coronavirus infection [24,25,26,27]. A viral miRNA acting to suppress or reduce AP-1 activity would possibly represent an advantage for the virus by halting in part the interferon mediated innate immune response by the cell.

Interestingly, using RNAhybrid software, we found that FOS is a predicted direct target of miR-AS1 (Figure 3B). To verify whether miR-AS1 targets FOS, we measured by Western blot c-FOS protein abundance in both HCT116 and HEK293T cells transfected with a synthetic miR-AS1 RNA oligonucleotide as compared to control sRNA transfected cells (Figure 3C). Our results confirm that miR-AS1 is able to significantly reduce c-FOS abundance in both cell lines.

To further confirm that miR-AS1 is able to down-modulate AP-1 activity, we measured AP-1 activity by a luciferase reporter assay [28] miR-AS1- transfected cells. In both HCT116 and HEK293T cells, transfection of miR-AS1 oligonucleotide significantly repressed AP-1 reporter luciferase activity compared to control unrelated RNA oligonucleotide (Figure 3D).

## 3. Methods

### 3.1. NGS Data Analysis

miRNA-seq raw reads (GEO GSE148729) were retrieved from NCBI SRA. Reads were trimmed with cutadapt v3.5 [29] to remove adapters, polyA tails added at 3′ ends (as a step of the library generation protocol) and three nucleotides (nts) at 5′ end (introduced by “template switch” library protocol). Trimmed reads were aligned to the hg38 human genome assembly with bowtie2. Reads without any valid alignment were aligned to the SARS-CoV-2 genome. Mapped reads were filtered with samtools [30] to remove reads longer than 25 nts and reads containing N cigar operations, which on the viral genome are generated by the “discontinuous extension” mechanism giving rise to viral sgRNAs [31]. Filtered reads were merged (bedtools merge) [32] and clusters ranging from 20 to 30 nts in length and attaining a coverage >20 reads were selected as candidate microRNAs. For each candidate region (plus 30 upstream and 30 downstream nts), a putative RNA secondary structure was computed with RNAfold [17] and an arbitrary threshold of −20 Kcal/mol MFE was set. Only reads clusters lying on a single arm of the predicted structure (i.e., overlapping a set of consistently oriented “brackets” in the “dot and brackets notation”) and having >60% of nucleotides paired in secondary structure were retained.

Read coverage on the SARS-CoV-2 genome was computed with bedtools genomeCoverage [32], scaled by the Million reads mapping on the SARS-CoV-2 genome, and visualized on UCSC genome browser [33].

### 3.2. Cell Lines and Reagents

Vero E6 cells (ATCC CRL-1586) were maintained in Modified Eagle Medium (MEM, Sigma Aldrich, St. Louis, MO, USA, M2279) supplemented with 10% heat inactivated fetal bovine serum (Gibco, Waltham, MA, USA, 10270), 2 mM L-glutamine, and 1% penicillin/streptomycin solution (Sigma Aldrich St. Louis, MO, USA, G7513; P0781) at 37 °C in a humidified atmosphere of 5% CO_2_. Calu-3 (ATCC HTB-55) were maintained in RPMI-1640 MEDIUM (Sigma Aldrich, R0883) supplemented as for MEM. Cells were exposed to SARS-CoV-2 isolate (2019-nCoV/Italy-INMI1, available from EVAg, Ref-SKU: 008V-03893, and from GISAID accession number EPI_ISL_410546) in medium without FBS for 1 h at 37 °C at a multiplicity of infection (MOI) 0.1 OR 0.01. At the end of the adsorption period, cells were washed and incubated in medium with 2% FBS, and where indicated, treated with enoxacin (Merck Life Science 557305) at the indicated concentrations.

HEK293T cells were grown in high glucose DMEM supplemented 10% (*v/v*) fetal bovine serum, 2 mM L-glutamine, and penicillin/streptomycin. HCT116 cells were grown in McCoy’s medium supplemented with 10% (*v/v*) fetal bovine serum (Euroclone, Milan, Italy), 2 mM L-glutamine (Euroclone ECB3000D), and penicillin-streptomycin (Euroclone ECB3001D). DNA and RNA oligonucleotides were purchased from Merck Life Science (Darmstadt, Germany), sequences are listed in Supplementary Table S1. Monoclonal rabbit antibody raised against FOS (clone 9F6 #2250) and monoclonal rabbit antibody raised against GAPDH (clone 14C10, #2118) were purchased from Cell Signaling Technologies. Monoclonal mouse antibody raised against *β*-tubulin (clone SAP 4G5 #T7816) was purchased from Sigma Aldrich. Goat Anti-Rabbit (H+L)-HRP Conjugate (BioRad #170-6515, Hercules, CA, USA) and Goat Anti-Mouse IgG (H+L)-HRP Conjugate (BioRad #170-6516) were used as a secondary antibodies. 3xAP1pGL3 (3xAP-1 in pGL3-basic) was a gift from Alexander Dent (Addgene plasmid # 40342; http://n2t.net/addgene:40342; RRID:Addgene_40342, accessed on 4 April 2023).

### 3.3. qRT-PCR

miRNA quantitative PCR and data analysis were performed as described previously [34,35]. Primer sequences are reported in Supplementary Table S1. Briefly, 500 ng of total RNA were reverse transcribed at 50 °C using Protoscript II Reverse transcriptase (#0368 New England Biolabs) according to the manufacturer’s protocol. As previously described, in order to avoid amplification of pre-miRNAs, RNA was not denatured before miRNA reverse transcription [34,35]. cDNA was amplified by qPCR using specific primers with GoTaq^®^ qPCR Master Mix (#6002 Promega, Milan, Italy) in a CFX Connect instrument (BioRad).

mRNA reverse transcription was carried out at 42 °C using Protoscript II Reverse transcriptase ( New England Biolabs GmbH #0368, Frankfurt, Germany) and random hexamers (Takara Bio Europe #3801, Saint-Germain-en-Laye, France) according to the manufacturer’s protocol. qPCR amplification of mRNAs was performed using specific primers with GoTaq^®^ qPCR Master Mix (#6002 Promega) in a CFX Connect instrument (BioRad).

### 3.4. miR-AS1 Target Prediction

To identify putative targets of miR-1AS, we run RNAhybrid software [23] on the human 3′ UTR sequences retrieved from the Targetscan v8 3′ UTR database [36,37]. RNAhybrid was run with the following parameters: −f 2,7 (to enforce miRNA-like seed pairing) −e 10 −p 0.5. Parameters fed to −d option were computed by running RNAcalibrate on the entire human UTR database with miR-AS1 sequence as query.

### 3.5. Statistical Analysis

Statistical data analysis was performed using the R software environment [38]. *p*-values to verify induction of miR-AS1 in infected cells or increased expression in enoxacin-treated cells were computed using a one-tailed *t*-test (for normally distributed data) or wilcoxon sum-rank test (for data not following a normal distribution). Whenever not specified, at least three independent replicates were analyzed.

### 3.6. Western Blot

Cells were harvested by centrifugation and resuspended in lysis buffer (NaCl 150 mM, Tris HCl pH 8 50 mM, EDTA 2 mM, NP-40 0.5%, glycerol 10%, protease inhibitor cocktail (Sigma Aldrich#P8340). A total of 40 ug of total protein extract were loaded on a 12% SDS PAGE gel and gels were blotted by semi-dry protein transfer to 0,45 µm nitrocellulose membrane (GE10600002 Amersham Protran). After membrane blocking with 5% (*v/v*) non-fat milk (A0830 PanReac Applichem) in TBS-tween 0.1%, membranes were incubated with specific antibodies and developed with chemiluminescence reagents (Supersignal West Pico PLUS Chemiluminescent Substrate Thermo Scientific (Waltham, MA, USA) 34577 for GAPDH and *β*-tubulin Western blot; Immobilon Western Merck WBKLS0500 for FOS Western blot). Images were acquired with Chemidoc MP Imaging System Bio-rad.

### 3.7. Luciferase Assays

A total of 250,000 cells were seeded in a well of a 24-well plate. For HEK293T cells, each well was transfected with 50 nM miR-1AS mimic (or control small RNA) using Interferin as transfection reagent (Polyplus 409). After 30 h, 250 ng (HCT116) or 100 ng (HEK293T) 3xAP1pGL3 reporter and 25 ng (HCT116) or 10 ng (HEK293T) pRL-TK (Promega E2241) plasmid were transfected using Jetprime transfection reagent (Polyplus 115). Eighteen hours later renilla and firefly luciferase activities were quantified using the Dual Glo Luciferase Assay kit (Promega E2920) according to the manufacturer’s instructions.

## 4. Discussion

We describe here a viral microRNA encoded by the minus strand of SARS-CoV-2, namely miR-AS1. Our data show that this miRNA is able to reduce host c-FOS protein expression levels, thus down-modulating AP-1 activity in human cells. Overall, our data suggest that this miRNA may contribute to viral replication by dampening AP-1 mediated immune response.

The region of the SARS-CoV-2 genome giving rise to miR-AS1 is very poorly conserved in both bat and vertebrate coronaviruses (Figure 1A), suggesting that the emergence of this small RNA may be a specific feature of SARS-CoV-2, not shared by other coronaviruses. Accordingly, our analysis pipeline applied to the twin datasets obtained by infecting Calu-3 cells with SARS-CoV-1 and MERS-CoV [16] did not highlight any putative miRNA in the corresponding regions of the SARS-CoV-1 and MERS-CoV genomes.

We speculate that the presence of miR-AS1 and miR-O7a [14,15] in the genome of SARS-CoV-2 may provide a rationale for the reported repressive effect of enoxacin on viral replication [21]. In fact, the positive strand of the SARS-CoV-2 genome may either serve as a mRNA and template for minus strand synthesis or be processed to yield miR-O7a [14]. Correspondingly, the negative strand of SARS-CoV-2 may either serve as a template for sgRNA and positive strand synthesis or be processed to yield miR-AS1. Unbalancing of this equilibrium by enoxacin towards the miRNA processing may substantially reduce the abundance of both strands of the viral genome, thus negatively affecting virus replication. Further investigation will be required to validate this hypothesis.

Before miR-AS1 can be expressed in infected cells, translation of the positive strand is needed to yield viral RNA polymerase which, in turn, is required for negative strand synthesis. Furthermore, negative polarity sgRNAs are expected to lack the region corresponding to ORF1ab (and hence miR-AS1 hairpin) which is only present in the full-length negative strand. Coherently, miR-AS1 is expressed at later stages of the infection (Figure 1). Notably, in Figure 1B miR-AS1 reads are normalized by the million reads mapping on the SARS-CoV-2 genome, thus subtracting the effect of the increasing number of infected cells (and viral genomes) which are expected to increase over a time-course. Nevertheless, miR-AS1 expression steadily increases over the course of the infection, thus confirming that miR-AS1 is a putative “late” gene in the infection process.

The exact role of AP-1 induction during coronavirus infection of human cells is still poorly understood. On the one hand, AP-1 induction is an essential step in the host response to the virus [39], on the other hand, it has been suggested that AP-1 induction may be triggered by alpha- and gamma-coronaviruses to enhance viral replication [26]. We, therefore, speculate that, after a transient induction of AP-1 in the first phases of infection, miR-1AS could act at later stages of the infection to limit AP-1 activity, possibly to reduce the host immune response to the infection. Further investigation will be required to ascertain the exact role of AP-1 induction and repression in the virus life cycle.

## Data Availability

Publicly available datasets were analyzed in this study. The dataset can be found here: NCBI GEO, accession number: GSE148729.

## Supplementary Materials

The following supporting information can be downloaded at: https://www.mdpi.com/article/10.3390/ncrna9030033/s1, Figure S1: A region of the SARS-CoV-2 genome ~300 nt apart of miR-AS1 and folding as as a hairpin but yielding no NGS reads does not yield any small RNA detectable by qPCR; Figure S2: RdRP expression in Calu-3 cells infected with SARS-CoV-2 in the presence of increasing concentrations of enoxacin.
