# Supplementary material for: A microRNA Arising from the Negative Strand of SARS-CoV-2 Genome Targets FOS to Reduce AP-1 Activity
Source: Noncoding RNA. 2023 May 23;9(3):33. doi: 10.3390/ncrna9030033 (PMC10301948; doi:10.3390/ncrna9030033)
Supplement: Supplementary file 1 [file ncrna-09-00033-s001.zip › ncrna-2358329-supplementary.pdf]

Supplementary Figure 1

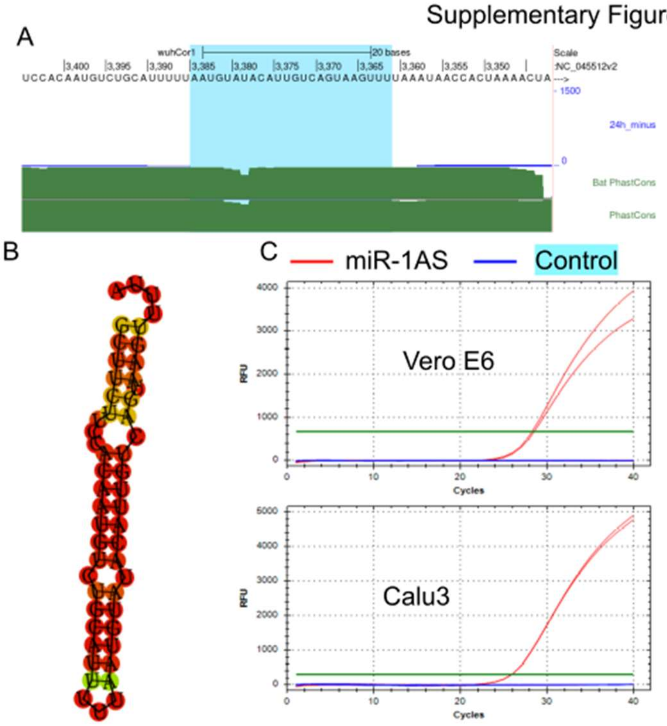

Figure S1: (**A**): Negative control locus on the UCSC genome browser. Blue track: miRNA-seq coverage (negative strand) normalized by SARS-CoV-2 mapping reads (RPM) at 24 hrs after infection of Calu-3 cells (GEO GSE148729 data). Green tracks: phastCons score across 44 bat coronaviruses (Bat Phastcons) and 119 vertebrate coronaviruses (Phastcons). (**B**): putative structure of pre-miR-AS1 computed using RNAfold. Color scale: base-pair probabilities. ©: qPCR amplification plots for miR-AS1 (red line) and negative control (blue line) in Vero E6 and Calu3 SARS-CoV-2 infected cells.

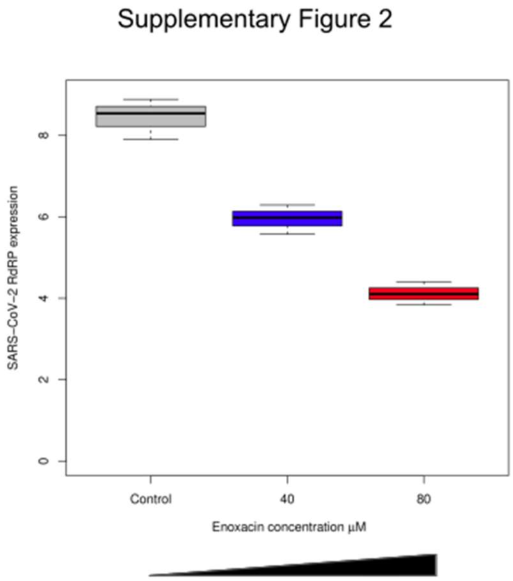

Figure S2: Enoxacin negatively affects SARS-CoV-2 replication in Calu3 cells. SARS-CoV-2 was assessed by qPCR amplification of RdRP viral gene, normalized against GAPDH (Arbitrary Units)
